# Deletion of a Seminal Gene Cluster Reinforces a Crucial Role of SVS2 in Male Fertility

**DOI:** 10.3390/ijms20184557

**Published:** 2019-09-14

**Authors:** Miyuki Shindo, Masafumi Inui, Woojin Kang, Moe Tamano, Cai Tingwei, Shuji Takada, Taku Hibino, Manabu Yoshida, Kaoru Yoshida, Hiroshi Okada, Teruaki Iwamoto, Kenji Miyado, Natsuko Kawano

**Affiliations:** 1 Division of Laboratory Animal Resources, National Research Institute for Child Health and Development, 2-10-1 Okura, Setagaya, Tokyo 157-8535, Japan; 2 Laboratory of Animal Regeneration Systemology, Department of Life Sciences, School of Agriculture, Meiji University, 1-1-1 Higashimita, Kawasaki, Kanagawa 214-8571, Japan; 3 Department of Reproductive Biology, National Research Institute for Child Health and Development, 2-10-1 Okura, Setagaya, Tokyo 157-8535, Japan; 4 Department of Systems BioMedicine, National Research Institute for Child Health and Development, 2-10-1 Okura, Setagaya, Tokyo 157-8535, Japan; 5 Laboratory of Regulatory Biology, Department of Life Sciences, School of Agriculture, Meiji University, 1-1-1 Higashimita, Kawasaki, Kanagawa 214-8571, Japan; 6 Faculty of Education, Saitama University, 255 Shimo-Okubo, Sakura, Saitama, Saitama 338-8570, Japan; 7 Misaki Marine Biological Station, Graduate School of Science, University of Tokyo, 1024 Koajiro, Miura, Kanagawa 238-0225, Japan; 8 Faculty of Biomedical Engineering, Toin University of Yokohama, 1614 Kurogane, Aoba, Yokohama 225-8503, Japan; 9 Department of Urology, Dokkyo Medical University Koshigaya Hospital, 2-1-50 Minamikoshigaya, Koshigaya, Saitama 343-8555, Japan; 10 Division of Male Infertility, Center for Human Reproduction, Sanno Hospital, International University of Health and Welfare, 8-10-16 Akasaka, Minato, Tokyo 107-0052, Japan

**Keywords:** gene cluster, deletion, semen, seminal vesicle proteins, male fertility

## Abstract

Multiple genes, whose functions or expression are overlapping, compensate for the loss of one gene. A gene cluster in the mouse genome encodes five seminal vesicle proteins (SVS2, SVS3, SVS4, SVS5, and SVS6). These proteins are produced by male rodents and function in formation of the copulatory plug following mating. SVS2 plays an essential role in the successful internal fertilization by protecting the sperm membrane against a uterine immune attack. We hypothesized that the four remaining seminal vesicle proteins (SVPs) of this gene cluster may partially/completely compensate for the deficiency of SVS2. For confirming our hypothesis, we generated mice lacking the entire SVP-encoding gene cluster and compared their fecundity with *Svs2*-deficient (*Svs2^−/−^*) mice; that is, mice deficient in *Svs2* alone. A single loxP site remained after the deletion of the *Svs2* gene. Therefore, we inserted another loxP site by combining the CRISPR/Cas9 system with single-stranded oligodeoxynucleotides (ssODN). Male mice lacking the entire SVP-encoding gene cluster (*Svs2–6^−/−^* mice) and thereby all five SVP proteins, generated by the deletion of 100kbp genomic DNA, showed low fecundity. However, the fecundity level was comparable with that from *Svs2^−/−^* male mice. Our results demonstrate that SVS3, SVS4, SVS5, and SVS6 do not function in the protection of sperm against a uterine immune attack in the absence of SVS2. Thus, *Svs2* is the critical gene in the SVP gene cluster.

## 1. Introduction

A gene cluster is a set of multiple tandemly aligned genes that comprise homologous sequences and often encode proteins with similar functions. For example, the discovery of the homeobox (Hox) gene clusters revealed that body segments emerge and are multiplied via a mechanism conserved from insects to mammals, including humans [1]. On the contrary, a bacterial operon contains several genes and their harmonized regulatory system works under the control of a single promoter [2]. Gene clusters are believed to originate from a single ancestral gene, however the molecular basis underlying their formation and the biological significance of this process is largely unclear.

The semen, a mixture of fluids secreted from the male accessory reproductive organs, seminal vesicles, prostate gland, and bulbourethral glands, contributes to critical events during internal fertilization. Proteins contained in the semen (seminal proteins) rapidly diverge by adaptive evolution [3,4,5,6]. In primates, the semenogelins (SEMGs), which are seminal vesicle proteins (SVPs), constitute the principal structural components of semen coagulum in the female reproductive tract [7]. The gain of the gene coding *SEMGII* is correlated with species-specific semen coagulum and with levels of female promiscuity [8].

**Figure 1:**
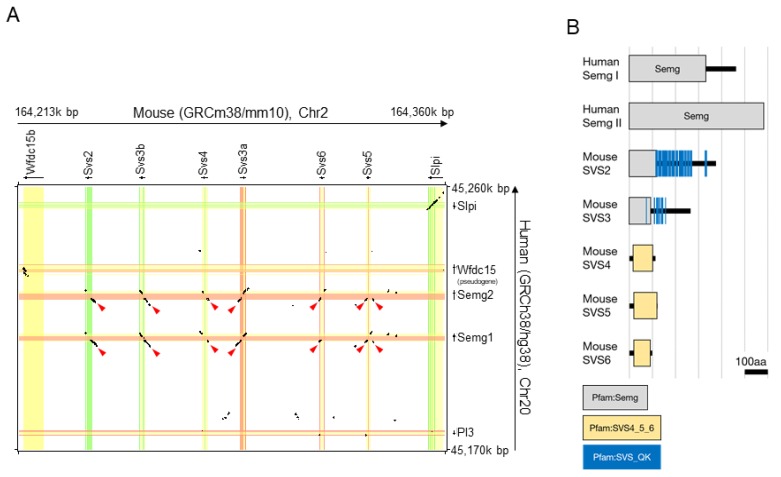
Comparative analysis of the mouse and human seminal proteins. (**A**) Dot-plot display of the mouse genome, including *Svs2-6* (*x*-axis), and the human genome, including *SemgI* and *II* (*y*-axis). Nucleotide positions are indicated for the mouse sequence on the *x*-axis, and sequence identity with the corresponding human sequence (50–100%) is shown as black dots. Shading in orange and green indicates the locations of exons on the + and – strands, respectively. Yellow areas represent introns. Red arrow heads indicate that the human *SemgI* and *II* share gene similarities with mouse *Svs2-6*. (**B**) Domain architectures of the human SEMGI and II and the mouse SVS2–6. SEMGI and II, as well as SVS2 and 3 share a SEMG domain (gray box). SVS4–6 share other domains (yellow box).

In rodents, the seminal vesicles secrete seven proteins, SVS1 to SVS7 [5]. As depicted in Figure 1A, *Svs2* is localized in mice on chromosome 2 and the five genes (*Svs3a, Svs3b, Svs4, Svs5,* and *Svs6*) encoding components of seminal vesicle secretions (SVS3, SVS4, SVS5, and SVS6) are tandemly aligned with *Svs2* in a gene cluster that is ~100 kbp long. The paralogous *Svs2–6* genes are suggested to duplicate from a gene that originated from a whey acidic protein four-disulfide core (*WFDC)*-type proteinase gene ancestor, as well as from *SEMGs* [9,10]. Previously, we showed that SVS2 inhibits sperm fertility as well as human SEMGs [11]. SVS2 prevents cholesterol-efflux from the murine sperm membrane and incorporates free cholesterol into the sperm membrane [12]. These results show that SVS2 suppresses the release of sterols from the sperm plasma membrane and plays a key role in unlocking sperm capacitation. Moreover, SVS2 is required for survival of sperms in the female reproductive tract [13]. Based on their identical genomic loci and partial similarity of nucleotide sequences, the *Svs2* to *Svs6* genes in mice are thought to correspond to human *SEMG1* and *II* genes (Figure 1A). Mouse SVS2 and SVS3 proteins share the SEMG domain with human SEMGI and II, albeit partially and only at the N-terminus (Figure 1B). SVS4, 5, and 6, with the SVS4_5_6 domain and without a SEMG domain, have a comparable effect as that of SVS2 and can compensate for the function of SVS2 [12]. The formation of a gene cluster often bestows similar, but possibly also distinct functions to genes such as those belonging to the Hox gene cluster. In this study, we generated mice lacking six serial (tandem) genes that encode SVPs to explore the role of a gene cluster in the formation of seminal vesicles and thereby male fertility.

## 2. Results

### 2.1. Designing gRNA for Deletion of a Cluster of Svs2–6 Genes

**Figure 2:**
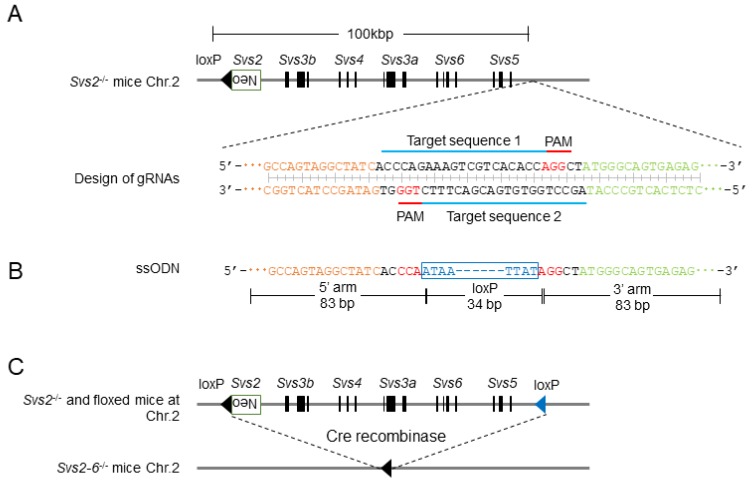
Genome editing strategy for knockin mice carrying a loxP cassette and knockout mice lacking five *Svs* genes. (**A**) Schematic representation of the Cas9-gRNA targeting sites. Two types of gRNAs were designed downstream of *Svs5*. Blue lines label the CRISPR target sequence. Red characters indicate protospacer adjacent motif (PAM) sequences. Orange and green characters indicate the upstream and the downstream region of the targeting site, respectively. (**B**) ssODN sequence for generating knockin mice. Knockin ssODN containing a loxP cassette and two 83 bp homology sequences. Blue characters indicate loxP sequence. Orange and green characters indicate homology arm with 5’ and 3’ of gRNA targeting sequence, respectively. (**C**) Generation of mice lacking *Svs2–6* (almost 100 kbp) by a cre-recombination system.

**Figure 3:**
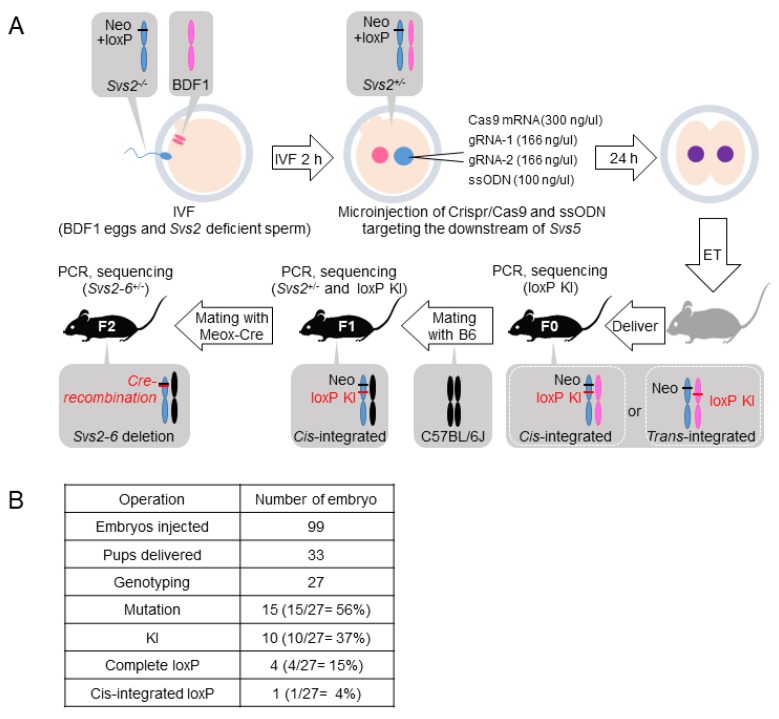
Experimental scheme for generating *Svs2-6^-/-^* mice. (**A**) Co-injection of gRNAs, Cas9 mRNA, and ssODN into fertilized BDF1 eggs with *Svs2*-deficient sperm. The loxP cassette should be cis-integrated with blue chromosomes carrying Neo-loxP from *Svs2^-/-^* mice. (**B**) Summary of the efficiency in CRISPR/Cas9 and ssODN induced mutations and loxP-knockin mice.

**Figure 4:**
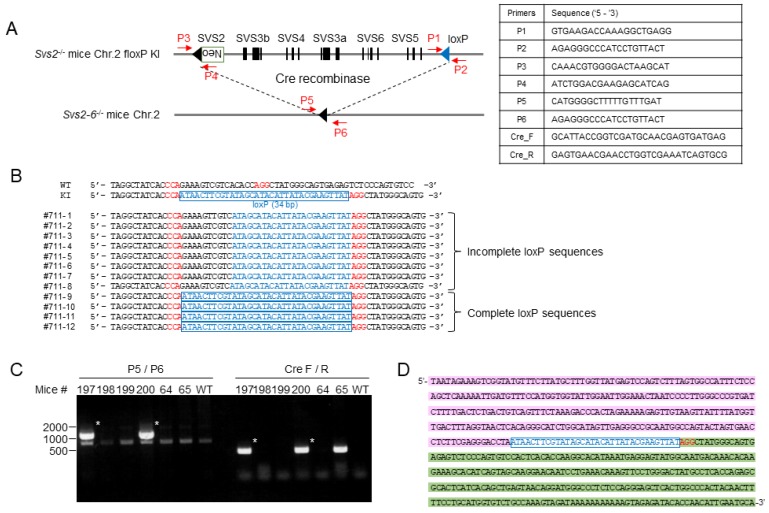
Validation of the *Svs2^−/−^* allele carrying a loxP-insertion using sequencing, PCR, and cre-recombination. (**A**) Primer sequences used in this study. (**B**) Sequence analysis of founder mouse (F0, #711). Genomic DNA of the #711 mouse was amplified by PCR (P1/P2) to detect loxP-insertion at the targeted locus. Genetic chimerism was observed in the #711 mouse; both complete and incomplete loxP sequences were detected in the mouse. (**C**) PCR analysis of F2 pups (#197–200, 64, 65) derived from crosses with F1 mice and *Meox-cre Tg* mice with primer sets P5/P6 as indicated in (**A**). PCR bands from *Svs2–6^+−-^* mice marked with asterisks display 1000 bp and are cre-positive. (**D**) Sequences of PCR products with using P5/P6 in an F2 mouse. Blue characters indicated loxP sequences, and red means PAM sequence. Pink and green shading characters indicate 5’ upstream of *Svs2* and 3’ downstream of *Svs5*, respectively.

*Svs2*-deficient (*Svs2^-/-^*) mice were generated as previously described [13]. Here, we generated additional loxP-knockin mice that lacked six serially placed genes (*Svs2*, *Svs3a*, *Svs3b*, *Svs4*, *Svs5*, and *Svs6*) (*Svs2-6*^−/−^ mice) by combining the CRISPR/Cas9 system and ssODN (Figure 2A–C). The Cas9 mRNA, gRNAs, and ssODN were microinjected into pronuclear eggs fertilized with sperms derived from *Svs2*^−/−^ mice in vitro (Figure 3A). We obtained 27 first generation (F0) pups from 99 injected embryos (Figure 3B). PCR amplification and sequencing revealed that 15 out of the 27 pups (56%) carried mutations at the target locus and 10 out of 27 pups (37%) tested positive for loxP cassette sequences. Six pups had a mutated loxP sequence and complete loxP sequences were detected in only four pups (15%). As shown in Figure 4B, one F0 male mouse (#711) had loxP knockin alleles, of which one was complete and another was a mutated loxP sequence. By mating this mouse with wild-type female mice, we generated second generation (F1) mice carrying a complete loxP sequence (Figure 3B). This F1 mice pool further comprised of two types of knockin mice: WT allele carrying a loxP cassette, and a *Svs2*-deficient allele carrying two loxP cassettes. The latter knockin mice, with *cis*-integrated-loxP, were further examined using PCR and sequenced with primers P1/P2 and P3/P4.

### 2.2. Generation of Svs2–6^−/−^ Mice

To obtain third generation (F2) mice carrying the heterozygous 100 kbp deletion, the F1 mice were mated with mice expressing a cre-recombinase (cre) under the control of a mesenchyme homeobox (*Meox*) gene (*Meox-cre* mice). Thus, we identified F2 mice carrying the 100 kbp deletion (*Svs2–6^+/−^*) by sequencing the combined sequences upstream of the *Svs2* gene and downstream of *Svs5* gene using primers P5/P6 and Cre_F/Cre_R (Figure 4C,D).

**Figure 5:**
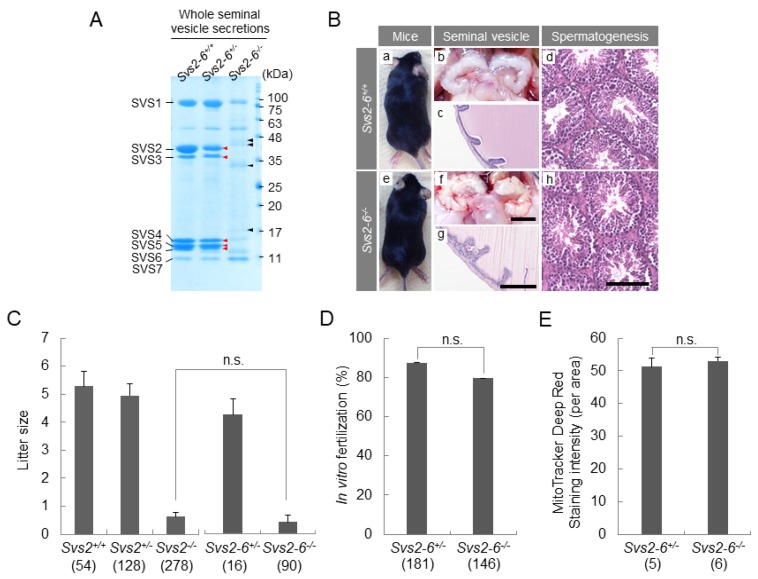
Reproductive phenotypes of *Svs2–6^−/−^* male mice. (**A**) CBB staining of the total inner fluid of seminal vesicle isolated from male mice. Red arrow heads: Proteins that disappeared/not detected in *Svs2-6^−/−^* male mice. Black arrow heads: Unknown proteins found in *Svs2-6^−/−^* male mice. (**B**) Representative pictures of 8-week-old *Svs2-6^+/+^* (a) and *Svs2-6^−/−^* (e) mice. Appearance of seminal vesicles collected from 8-week-old mice (b and f). Scale bar, 5 mm. Hematoxylin and eosin (H&E) stained sections of seminal vesicle tissue from 8-week-old mice (c and g). Scale bar, 100 μm. Testicular sections stained with H&E (d and h). Scale bar, 100 μm. (**C**) Fecundity of male mice. Parentheses, numbers of male mice examined. (**D**) The rate of fertilization of eggs with epididymal sperm collected from *Svs2-6*^+/−^ and *Svs2-6*^-/-^ mice. Values indicate mean ± SEM of triplicate experiments. Numbers in parentheses indicate the number of eggs examined. (**E**) Fluorescence intensity of mitochondrial staining in epididymal sperm from *Svs2-6*^+/*−*^ and *Svs2-6^−^*^/*−*^ mice. Parentheses, numbers of male mice examined.

To confirm the generation of mice lacking SVPs (SVS2, SVS3, SVS4, SVS5, and SVS6), we performed SDS polyacrylamide gel electrophoresis (SDS-PAGE) using lysates of seminal vesicle secretions collected from *Svs2-6^−/−^* male mice. The polyacrylamide gels were stained with coomassie brilliant blue (Figure 5A). The resolved proteins were comparably expressed in the seminal vesicle secretions collected from wild-type (*Svs2-6^+/+^*) and heterozygote (*Svs2-6^+/−^*) male mice. They were not detected in the seminal vesicle secretions collected from *Svs2–6^−/−^* male mice. Since the two genes that encode SVS1 and SVS7 are localized on chromosomes separated from a gene cluster encoding SVS2 to SVS6, we assumed that the expression of these two proteins would remain unaffected. Unexpectedly, the expression of SVS1 protein was reduced and unidentified proteins were detected in *Svs2-6^−/−^* male mice, compared with *Svs2-6^+/+^* and *Svs2-6^+/−^* mice. In contrast, the expression of SVS7 was unaffected in *Svs2-6^−/−^* male mice.

*Svs2–6*^−/−^ mice were born healthy and grew normally (Figure 5B). Despite being deficient in *Svs2, Svs3a, Svs3b, Svs4, Svs5,* and *Svs6,* the seminal vesicles were formed normally. This result suggests that a specific gene cluster encoding SVPs is not essential for the growth and differentiation of seminal vesicles.

### 2.3. Male Fertility of Svs2–6^−/−^ Mice

Our previous report showed that the fertility of *Svs2^−/−^* male mice was strongly reduced [13]. The fertility of *Svs2–6^−/−^* male mice was also reduced but was comparable with mice lacking a single *Svs2* gene (0.43+/−0.14 and 0.63+/−0.13, respectively; *p* = 0.420) (Figure 5C). Similar to *Svs2^−/−^* male mice, no copulatory plugs were formed inside the vagina of female mice that mated with *Svs2–6^−/−^* male mice, in spite of observed mating behavior and normal production of sperms in these mice (Figure 5*B*). In vitro fertilization (IVF) and a mitochondrial activity assay showed that the epididymal sperms of *Svs2–6*^−/−^ male mice were comparable to those of *Svs2–6*^+/^^-^ male mice (Figure 5D–E). It is known that hormonal condition is important for acquisition and maintenance of male fertility. Although we previously measured testosterone concentration in the serum of male mice [13,14], there were no significant differences due to individual variability but not genotypes. Therefore, we removed the results of hormonal measurements in this study. Otherwise, sex behavior, spermatogenesis, and sperm quality in *Svs2–6^−/−^* mice are comparable to that of *Svs2–6^+/+^* male mice, indicating that hormonal concentrations are normal in *Svs2–6^−/−^* mice.

On the basis of this, we concluded that the SVS3–6 proteins play no additive roles, at least in male fertility, and the formation of seminal vesicles. Thus, SVS2 is the sole key factor for regulating male fertility through seminal vesicle secretions.

## 3. Discussion

To gain a comprehensive understanding of the function of the *Svs* gene family, we generated *Svs2–6^−/−^* mice lacking the entire *Svs2–6* gene locus. Although epididymal sperms collected from *Svs2–6*^-/-^ mice were normal in morphology and quality, their fertility was quite low in the female reproductive tract (Figure 5C–E). Moreover, we observed no significant difference between the *Svs2–6^−/−^* mice and the *Svs2^−/−^* male mice generated in an earlier study [13], except that the fecundity with sperms from the *Svs2–6^−/−^* male mice tended to reduce slightly compared with those from the *Svs2^−/−^* male mice (Figure 5C). This result is consistent with our previous study that the ganglioside GM1, functional as a sperm receptor, interacts with SVS2 at a higher level than those of SVS3 and SVS4 [12]. Moreover, SVS2 proteins aid epididymal sperms achieve uterine survival without SVS3–6 [13]. Ramm et al. [5] used comparative proteomic techniques and reported that SVS2 correlates positively with sperm competition levels across species. The results of the current study in combination with literature evidences indicate the pivotal role of SVS2 in uterine sperm survival in seminal plasma. They point out that SVS3–6 proteins are unable to compensate for SVS2 deficiency in internal fertilization.

In natural mating, SVS2 is degraded in various length in the female reproductive tract [11]. Similarly, SEMG is degraded by the serine protease termed prostate-specific antigen (PSA) secreted from the prostate in humans [15]. From the fragmentation of SVPs in humans and mice, we hypothesize that SVS2 and SEMGI/II contain a functionally conserved domain with multiple or short sequences. Actually, SEMGI and II include repeated units and work as a sperm motility inhibitor and bactericidal agent after their degradation [16,17,18]. Although the mechanism of acquisition of SVS2 function is still unclear, the domain analysis reveals unique repetitive domains, SVS_glutamine and lysine (QK), which are repeated 17 times in the SVS2 sequence (Figure 1A). A single SVS_QK domain consists of 12 amino acids including basic amino acids K, and is detected only in SVPs derived from rats and mice in mammalian species. Since none of the SVS4, 5, and 6 have this domain, we assumed that the SVS_QK domain could be evolved after expansion of the *Svs* gene cluster. Although the function of the SVS_QK domain is unclear, the constant repeat of basic amino acids may enable immunological tolerance in the female reproductive tract and/or sperm protection against female immune system.

The *Svs* gene cluster emerged by a tandem duplication in recent evolution [5]. The genes consist of three exons, and the nucleotide sequences of the *Svs2*–*6* genes are highly similar with the human *SemgI* and *II* at the first exons and their upstream regions that contain predicted promoter regions (Figure 1A). Differences are observed in the second exon which encodes almost all of the secreted protein that serves in formation of a semen gel or clot (copulatory plug). The amino acid sequences of the secreted proteins encoded by *Svs2–6* genes are diverse and this diversification can be explained by the rapid evolution of the *Svs* gene family, which corresponds to adaptation against species-specific mating behavior and/or speciation [8,19]. In a similar case, several gene families such as genes encoding the major histocompatibility complex (MHC), antimicrobial peptides, and immunoglobulin typically have several paralogs and pseudogenized duplicated genes [20,21,22,23]. A group of MHC class I-like molecules, known as NKG2D ligands, are prime targets for immune evasion strategies deployed by many viruses [24]. An ancestral *NKG2DL* gene located in the MHC region underwent duplication, subsequently producing a *MIC* gene and *MILL* gene [25]. These two genes have been retained in the marsupial opossum, however the *MILL* gene was lost in humans, while the *MIC* gene was lost in rodents. The species-specific evolution of the *NKG2DL* gene family causes infection of the species-specific pathogen. From this immunological viewpoint, SVS2 proteins in mice mainly function in protection of sperms against female immune response in the uterus. The SVS3–6 proteins or further new duplications may serve as an adaptor response against the evolution of female spermicide and sperm receptors against SVS2.

Eukaryotic genes are considered to be independently expressed under the control of their own promoters and cis-regulatory elements. As mentioned above, genes co-habited on a chromatin domain or genomic neighborhood are co-expressed frequently, but these co-expressed genes are often free from functional partnership within the same biological phenomena [26]. Although the mechanisms for partitioning the genome into clusters of co-expressed genes are poorly understood, their conservation of clustering across species suggests that this organization did not arise randomly but selectively. Therefore, the perturbation of such an organization may prove deleterious for the continued existence of a species. In the context of human diseases, dysregulation of gene expression across genomic neighborhoods causes highly pleiotropic diseases, including genomic instability disorders, global covalent histone modifications, and DNA methylation. In case of seminal vesicle secretions in *Svs2–6^−/−^* mice, we observed an increased expression of unknown proteins, which could not be detected in *Svs2–6^+/−^* mice (Figure 5A). This implied that the deletion of a seminal gene cluster in mice could disturb genomic neighborhoods, presumably leading to short-time speciation in vitro.

In conclusion, we succeeded in generating *Svs2–6^−/−^* mice with a 100 kbp genomic deletion by combining CRISPR/Cas9 system with ssODN. We confirmed that SVS2 plays the major role in male fertility by functioning as a component of the murine seminal plasma. Present diagnosis of male infertility is made by sperm number and quality but seminal plasma proteins surrounding the sperm, because the importance of seminal plasma proteins in internal fertilization is ignored in humans. Our results raise the possibility that lack of a seminal protein, i.e., human SEMGI/II, may cause male infertility in humans. In near future, the evaluation of seminal plasma proteins (quantity and quality) may be added to the diagnosis check list of infertile patients.

## 4. Materials and Methods

### 4.1. Production of Knockout Mice

*Svs2*-deficient (*Svs2^−/−^*) mice were generated as previously described [13]. Here, we produced mice lacking six serial genes (*Svs2*, *Svs3a*, *Svs3b*, *Svs4*, *Svs5*, and *Svs6*) (*Svs2–6^−/−^* mice) by combining the CRISPR/Cas9 system with single-stranded oligodeoxynucleotides (ssODN). Software tools (crispr.genome-engineering.org), which predict unique target sites that recognize the downstream of *Svs5* throughout the mouse genome, were used for designing gRNAs (Figure 2A). The 19 base sequences were cloned into a modified gRNA cloning vector (Addgene plasmid ID 41824, Watertown, MA, USA) and served as templates for in vitro transcription using mMassagemMachine T7 kit (Thermo Fisher Scientific Inc., Waltham, MA, USA) as previously described [27]. ssODN included homology arms (83 bp) to link the double strand break of the original mouse DNA, and a 34 bp loxP cassette sequence was purchased from Integrated DNA Technologies Inc. (Coralville, Iowa, USA) (Figure 2B).

Eggs were collected from the BDF1 strain of superovulated female mice (Figure 3A). ssODN with gRNAs and Cas9 mRNA prepared by hCas9 expressing vector (Addgene, plasmid ID 41815) were microinjected into pronuclear eggs fertilized with the sperms derived from *Svs2^−/^^−^* mice. Embryos that were developed to the two-cell stage were transferred to pseudo-pregnant ICR female mice. F0 generation mice were genotyped by PCR amplification using primers P1/P2 and the sequencing of inserted loxP (Figure 4A). F1 mice (*Svs2^+/^^−^*, loxP-KI) produced by crossing F0 with C57BL/6, were genotyped by PCR and sequenced by using primers P1/P2 for detecting the inserted loxP and primers P3/P4 for confirming *Svs2* deficiency (Figure 4A). F2 mice (*Svs2-6^+/^^−^*) generated by crossing F1 with B6; 129S4-Meox2<tm1(cre)Sor>/J (*Meox-cre* Tg mice) [28], were genotyped by PCR and sequenced using primers P5/P6 and Cre_F/R (Figure 4A). We defined the *Svs2–6^−/^^−^* mice as those bearing a deletion of 100 kbp including *Svs2*, *Svs3a*, *Svs4*, *Svs3b*, *Svs6,* and *Svs5* on chromosome 2.

All mice were housed in specific pathogen-free controlled conditions. Food and water were available ad libitum. The procedures for performing animal experiments were approved by The Institutional Animal Care and Use Committee of the National Research Institute for Child Health and Development (1/April/2004).

### 4.2. Preparation of Seminal Vesicle Secretions

Seminal vesicles were isolated from 8- to 20-week-old male mice, and the fluids extracted from the seminal vesicles were dissolved completely in 1 mL of 8 M urea. Each sample was separated by SDS-PAGE and stained with coomassie brilliant blue (CBB) as previously described [11]. All experiments were performed with the approval of the Animal Care Committee of the Meiji University (IACUC15-0014).

### 4.3. Male Fertility In Vivo and In Vitro

To evaluate male fertility in vivo, the number of pups delivered by 8- to 16-week-old female mice were recorded after a 2-week mating period, during which two female mice were housed with a single 8- to 16-week-old male mouse. 

For *in vitro* fertilization (IVF), eggs were collected from superovulated C57BL/6J female mice (8–12 weeks old) 14–16 h after human chorionic gonadotropin (hCG) injection [13]. The sperm collected from the epididymides of 12- to 24-week-old male mice were capacitated by incubating in HTF medium for 2 h before insemination. The final concentration of the sperm added to the eggs was 1.5 × 10^5^ sperm/mL.

Sperm mitochondrial activity *in vitro* was evaluated by staining with MitoTracker Deep Red FM (Thermo Fisher Scientific Inc., Waltham, MA, USA) as described previously [29]. Epididymal sperms from male mice were incubated in medium containing 500 nM MitoTracker Deep Red FM for 30 min at 37 °C and observed using a confocal laser microscope (LSM510Meta: excitation at 633 nm and emission at 650 nm). 

Data are presented as mean values ± standard error of the mean. The Student’s t-test was used for statistical analysis; significance was assumed at *p* < 0.05.
